# Development and validation of the Klinefelter-Associated Neurodevelopmental Difficulties (KAND) Checklist: a three-phase mixed-methods study

**DOI:** 10.1186/s11689-025-09670-0

**Published:** 2026-01-30

**Authors:** Kaat Theelen, Costantino Galasso, Wilfried Cools, Inge Gies, Stephanie Vanclooster, Anna C. Jansen

**Affiliations:** 1 Faculty of Medicine and Pharmacy, Vrije Universiteit Brussel, Brussels, Belgium; 2 Centre for Rare Diseases, Universitair Ziekenhuis Brussel, Brussels, Belgium; 3 Support for Quantitative and Qualitative Research (SQUARE), Vrije Universiteit Brussel, Brussels, Belgium; 4 Division of Pediatric Endocrinology, Department of Pediatrics, Universitair Ziekenhuis Brussel, Brussels, Belgium; 5 Vitality Research Group, Vrije Universiteit Brussel, Brussels, Belgium; 6 Mental Health and Wellbeing Research Group, Department of Health Sciences, Vrije Universiteit Brussel, Brussels, Belgium; 7 Centre for Medical Genetics, Antwerp University Hospital, Antwerp, Belgium; 8 Division of Pediatric Neurology, Department of Pediatrics, Antwerp University Hospital, Antwerp, Belgium; 9 Translational Neurosciences, University of Antwerp, Antwerp, Belgium

**Keywords:** Klinefelter syndrome, Neurodevelopmental difficulties, Psychosocial functioning, Participatory research, Self-report tool, Multidisciplinary care, Checklist development

## Abstract

**Background:**

Klinefelter syndrome (KS) (47,XXY) is a genetic condition associated with infertility, hormonal imbalances, and a range of neurodevelopmental and psychosocial challenges, including language impairments, executive dysfunction, and difficulties in social functioning. Although these challenges can significantly affect daily life, no uniform terminology or standardized tool currently exists to systematically identify and assess them. Building upon research in tuberous sclerosis complex (TSC) and using the TSC-Associated Neuropsychiatric Disorders (TAND) Checklist as a foundation, this study explored neurodevelopmental difficulties associated with KS and led to the development, initial validation, and translation of the Klinefelter-Associated Neurodevelopmental Difficulties (KAND) Checklist.

**Methods:**

A three-phase mixed-methods design was used. In Phase 1, a literature review and semi-structured interviews with nine adults with KS and eleven parents identified relevant challenges and informed the preliminary checklist (KAND-PL). In Phase 2, feedback from twenty-four parents and thirteen healthcare professionals, collected through a feedback form and focus group, guided iterative revisions to produce the pre-final version (KAND-PF). In Phase 3, the checklist was evaluated using participant feedback and exploratory psychometric analyses with eighteen individuals with KS and twenty-nine parents. Internal consistency (Cronbach’s α) within conceptually related item groups and correlations with validated behavioral measures (Spearman’s ρ) were examined. Thematic analysis and descriptive statistics were applied to qualitative and quantitative responses.

**Results:**

Building on the TAND Checklist, the KAND Checklist addresses KS-specific challenges across 13 domains and a background information section on discussing the diagnosis. Across all phases, input from individuals with KS, parents, and healthcare professionals provided strong evidence for face, content, and response process validity, demonstrating that the checklist covers relevant difficulties, uses clear language, and is feasible for self- and proxy-completion. Internal consistency was good, and preliminary convergent validity was supported through correlations with existing behavioral measures.

**Conclusion:**

The KAND Checklist is a reliable, accessible tool for identifying neurodevelopmental difficulties in KS. It is intended to raise awareness, facilitate structured clinical conversations, and encourage individualized care. This study highlights the value of participatory research in developing meaningful tools, promoting holistic care, and fostering collaboration between individuals with KS, their families, and professionals. Further dissemination, translation, and larger-scale validation are needed.

**Supplementary Information:**

The online version contains supplementary material available at 10.1186/s11689-025-09670-0.

## Background

Klinefelter syndrome (KS) (47,XXY) is a genetic condition in males characterized by an extra X chromosome. First described in 1942 in adults with infertility, hypogonadism, and gynecomastia, KS is now known to be associated with a broad range of neurodevelopmental and psychological difficulties, although their precise delineation remains unclear [1–5].

Studies on mood and behavioral issues in individuals with KS suggest an increased risk for internalizing problems, including anxiety, depression, somatic complaints, and social withdrawal, while externalizing difficulties appear comparable to the general population, apart from more frequent emotional outbursts and aggression linked to atypical emotional regulation [5–9].

Most individuals with KS have intellectual abilities ranging from average to below average, with a characteristic pattern of lower verbal than performance IQ [10]. Findings on neuropsychological abilities in individuals with KS are largely consistent. Executive functioning, attention, and cognitive flexibility are often affected with evidence suggesting that these challenges represent a core neuropsychological feature of KS rather than being secondary to co-occurring ADHD [4, 9, 11].

Language and social communication difficulties affect 70–90% of individuals with KS and include delays in receptive and expressive language, oral motor function, language-memory, literacy, and social-pragmatic skills [12, 13]. These difficulties along with deficits in social cognition - which are well documented in KS [4, 5, 14] - contribute to academic and daily-life challenges, emphasizing the importance of routine assessment of social-cognitive abilities [4, 15].

Regarding psychiatric disorders, a higher prevalence of ADHD symptoms and autistic traits are commonly reported, yet evidence for higher rates of formal diagnoses remains limited [7, 8, 16, 17]. Anxiety disorders also appear to be more frequent in KS, although some findings may be influenced by the absence of uniform family psychiatric histories [7].

The impact of KS on psychosocial functioning is clear: individuals with KS frequently report challenges with social interaction, including difficulties with peer contact, conflict resolution, social fluency, and emotional expression [8, 9]. The most frequent reported challenges in terms of personal impact of living with KS are infertility, body image concerns, psychological problems, and learning difficulties [18]. Physical features such as tall stature, gynecomastia, reduced muscle mass, small testes, and limited body hair can negatively affect self-esteem, body-image, quality of life, and increase the vulnerability to bullying [18–22].

In sum, scientific evidence shows that individuals with KS are at risk for a range of neurodevelopmental and (psycho)social difficulties. However, most studies have examined these domains in isolation, leading to a fragmented understanding that does not represent clinical reality. A more integrated understanding is needed to guide healthcare professionals to support individuals and families with KS in a holistic way, as they are confronted with both physical and non-physical obstacles [1, 3, 5, 9, 21–23]. A standardized tool containing uniform language to identify and discuss these difficulties could help bridge the gap between research and clinical practice.

Inspired by similar efforts in the tuberous sclerosis community [24, 25], this study introduces Klinefelter-associated neurodevelopmental difficulties (KAND) as an umbrella term to unify these challenges and provide a shared language to enhance awareness, understanding, and communication among healthcare professionals, individuals living with KS, and their families. In conjunction, we present the KAND Checklist: a self-report and proxy-report tool that is based on the framework of the validated TAND Checklist (Table 1), scientifically validated (Table 2) and developed in partnership with the KS community. The checklist serves as a screening and supporting tool only; it is not diagnostic or predictive in nature, and does not track changes or progression over time [26]. It is designed to help individuals with KS and their families identify individual strengths and challenges, and to support more effective communication about these issues with healthcare providers. Implementation of the checklist is expected to facilitate early recognition, appropriate support/treatment, and timely referral to specialists.

**Table 1:** Structure of the TAND Checklist (Self-report, Quantified version)

Item	Level of investigation
Question 1	Developmental milestones
Question 2	Current level of functioning
Question 3	Behavioral causing concern
Question 4	Psychiatric disorders
Question 5	Intellectual ability
Question 6	Difficulties in learning in school
Question 7	Difficulties in brain skills
Question 8	Psychosocial difficulties
Question 9	Additional concerns
Question 10	Parent, caregiver, or self-rating of the impact of TAND
Question 11	Prioritizing list
Question 12	Strategies
Question 13	Strengths
Question 14	TAND Cluster Profile

Adapted from de Vries et al. (2015) and Heunis et al. (2023) [24, 27]

## Methods

### Design and setting of the study

**Fig. 1:**
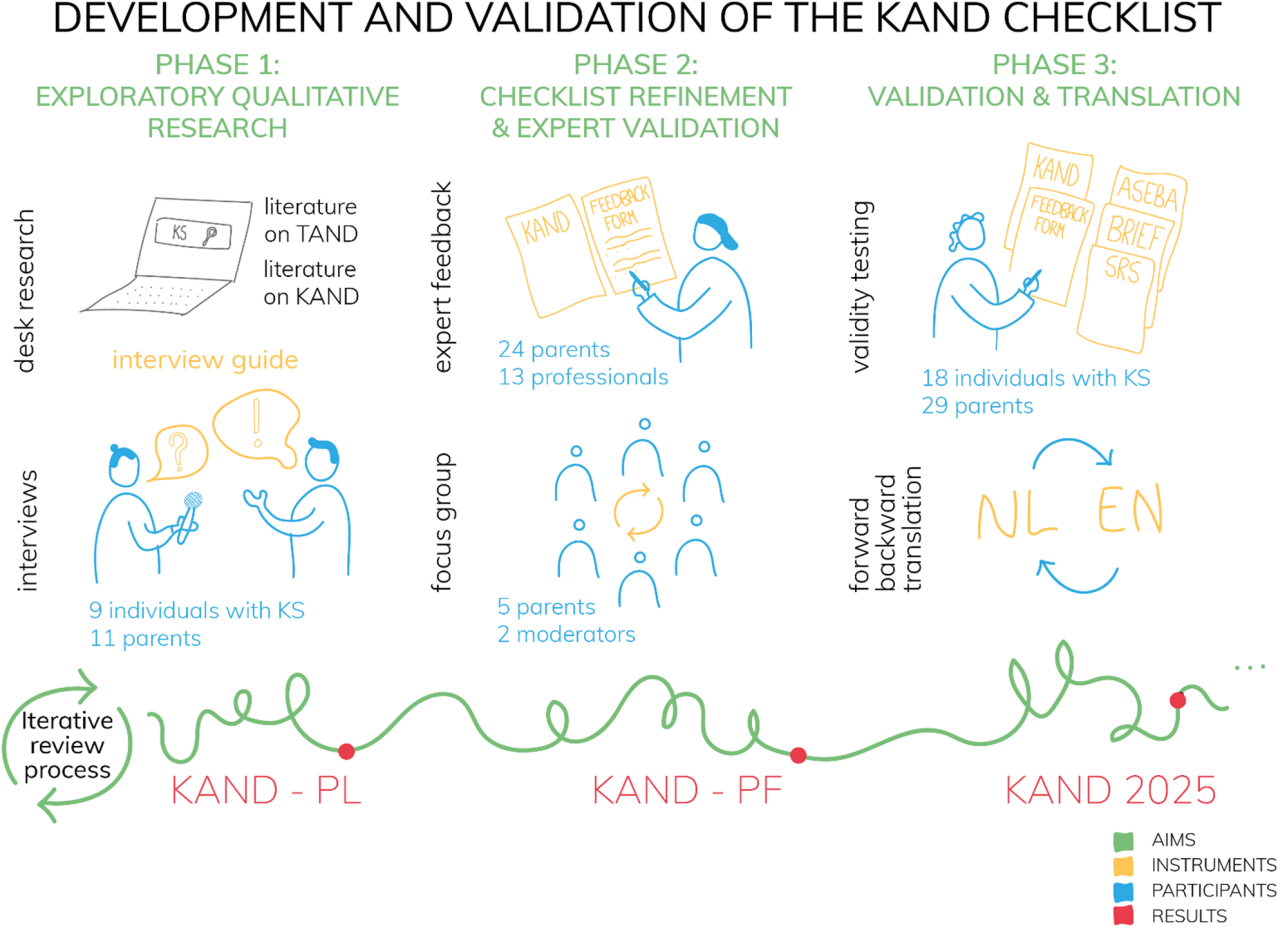
Visual summary of the study design used to develop and validate the KAND Checklist. KAND = Klinefelter-associated neurodevelopmental difficulties; TAND = TSC-Associated Neuropsychiatric Disorders; KAND-PL = KAND Checklist-Preliminary version; KAND-PF = KAND Checklist-Prefinal version; KAND 2025 = KAND Checklist (Final version 2025); ASEBA = Achenbach System of Empirically Based Assessment; BRIEF = Behavior Rating Inventory of Executive Function; SRS = Social Responsiveness Scale; NL = Dutch; ENG = English

The study used a multiphase mixed-methods design (Fig. 1) conducted in three sequential phases:

**Phase 1*** – Exploratory qualitative research:* A narrative literature review was conducted to inform the development of an interview guide. Using the guide, semi-structured interviews were conducted with individuals with KS and their parents to support the adaptation of the TAND Checklist to the needs of individuals with KS. Insights from literature and findings from the interviews were integrated to develop a preliminary version of the KAND Checklist (KAND-PL).

**Phase 2*** – Checklist refinement and expert validation:* A mixed-methods approach was used to collect qualitative and quantitative feedback from expert professionals and parents of individuals with KS. This feedback was used to iteratively refine the checklist, leading to a pre-final version (KAND-PF).

**Phase 3** *– Validation and translation:* The KAND Checklist was evaluated through a combination of qualitative and quantitative methods. Participant feedback was collected to assess face validity, content validity, and response process validity. Exploratory psychometric analyses were performed, including internal consistency within conceptually related item groups (KAND groups) and correlations with validated behavioral measures, to provide preliminary evidence for convergent validity. Based on these findings, a definitive version of the KAND Checklist (KAND 2025) was created. This was followed by a forward-backward Dutch to English translation process.

### Participant recruitment

Participants were primarily recruited through convenience sampling in collaboration with the Klinefelter Clinic of UZ Brussel, the Dutch Klinefelter Community, and the TRIXY Center of Expertise at the University of Leiden. A small number of additional participants were recruited using snowball sampling. Data collection took place at the participants’ homes, at the hospital or online. To be included in phase 1 of this study, subjects needed to be diagnosed with KS or be a parent of an individual with KS. In phase 2, subjects needed to be a parent of an individual with KS or a professional with expertise in Klinefelter care. In phase 3, subjects needed to be diagnosed with KS or be a parent of an individual with KS. Participants were required to be Dutch-speaking adults (≥ 18 years) capable of completing the checklist, feedback forms, and questionnaires, as well as participating in focus groups. There was no upper age limit.

### Materials

#### Interview guide (Phase 1)

Based on the existing literature on neurodevelopmental difficulties in KS and the framework of the TAND Checklist (Table 1), an interview guide consisting of 9 different topics was developed: health trajectory, behavioral concerns, psychological functioning and psychiatric problems, cognitive ability, school life and participation, neuropsychological skills, language and communication, self-perception and individual experiences and needs. The interview guide contained supporting questions presented to the respondents during the interviews. The interview guide was not pilot tested and is available upon request.

#### Feedback form (Phase 2 and 3)

A feedback form was developed to evaluate the KAND Checklist’s completeness, clarity, ease of use, clinical applicability, and likelihood of prompting referrals for further assessment. For each of these five topics, the form included a rating scale from 0 (very poor) to 4 (excellent), followed by a space for written comments to explain or elaborate on the given score (e.g., suggestions, remarks, or additions). The feedback form is available upon request.

#### Validated behavioral measures (Phase 3)

The validated behavioral measures were selected following phase 2, after approval of the amended protocol by the Research Ethics Board. The Behavior Rating Inventory of Executive Function (BRIEF) [28] is a questionnaire developed to quantify behavioral manifestations associated with executive functioning in children, adolescents, and adults. The Social Responsiveness Scale (SRS) [29] is a screening tool for autism spectrum disorder and quantifies difficulties with social behavior and communication. The Achenbach System of Empirically Based Assessment (ASEBA) [30] assesses abilities, emotional and behavioral problems. For each of the three questionnaires, we used adult self-reports, adult informant lists, and informant lists for minors: BRIEF adult version (BRIEF-A) self-report and informant report, BRIEF second edition (BRIEF-2) parent form, SRS adult version (SRS-A) self-report and informant report, SRS second edition (SRS-2) parent form, ASEBA Adult Self-Report version (ASR), ASEBA Adult Behavior Checklist (ABCL), and ASEBA Child Behavior Checklist (CBCL). These validated questionnaires were used to validate the KAND Checklist, which was completed in parallel. All materials were provided in Dutch.

### Ethical review and consent

This study was approved by the Research Ethics Board of UZ Brussel on 24 June 2020 (reference number: B1432020000124) and amended. Each participant was informed in advance about the objective and methodology of this study and provided formal written consent to be included. Confidentiality was maintained through anonymized data processing. This is not a clinical trial.

### Data collection

#### Phase 1

Between July 2020 and February 2021, data were collected through semi-structured interviews using an interview guide developed based on the narrative literature review. The review aimed to provide an overview of existing literature on various aspects of KAND and was guided by topics from the TAND framework. Most interviews took place at the participants’ homes. Owing to safety precautions during the COVID-19 pandemic, some interviews took place online. With participant consent, interviews were audiotaped for accurate transcription and analysis. The interviews lasted between 30 minutes and 2 hours 20 minutes. After each interview, the investigator made field notes with subjective information or personal thoughts that could bias the study or influence data interpretation. We aimed to reach data saturation.

#### Phase 2

Between October and December 2023, quantitative and qualitative feedback was collected on the preliminary version of the KAND Checklist from two expert groups: individuals with professional expertise in KS and parents with lived experience with KS. If individuals were interested in the study, we provided study details: to expert professionals by email and to expert parents during a face-to-face meeting or through a video call. When they agreed to be included, an informed consent form was signed, after which they received the preliminary version of the KAND Checklist in an electronic or paper format together with a feedback form.

Additional feedback was obtained through an online focus group, moderated by two independent researchers experienced in qualitative methods. All members of the expert parent group were invited via email. Parents consented to participate. The focus group aimed to deepen understanding of the main qualitative findings from phase 2 and to explore participants’ perspectives on the checklist’s relevance and usability. The focus group lasted 2 h 20 min. The discussion was audio recorded with participants’ permission and both moderators took independent field notes to capture non-verbal cues and contextual details that complemented the transcript data.

#### Phase 3

Between March and May 2024, quantitative and qualitative data were collected for validation of the KAND Checklist. A group of adult individuals with KS and parents of individuals with KS participated. Similar to the previous phase(s), the subjects were invited by phone, email, or video-call. All the participants were asked to complete five questionnaires (i.e., the KAND-PF, feedback form, BRIEF, SRS, and ASEBA) at their preferred locations. The researcher explained the different steps, provided the questionnaires, and answered questions. There was no time restriction to complete the questionnaires. All questionnaires were given back in person or sent back by (e)mail.

### Data analysis

#### Overview validity evidence

Table 2 provides an overview of the types of validity evidence collected throughout the study, along with the methods used for assessment and the corresponding phase in which each was gathered.

**Table 2:** Types of validity evidence and methods of assessment used in this study

Types of validity evidence	Definition	Method of assessment	P1	P2	P3
*Face validity*	Does the checklist measure the Klinefelter-associated difficulties?	Interviews Expert/user feedback	x	x	x
*Content validity*	Does the checklist measure all the components of relevance to KAND?	Interviews Expert/user feedback	x	x	x
*Response process validity*	Is the checklist easy to use, clearly understandable, and culturally appropriate for those who use it?	Expert/user feedback		x	x
*Internal consistency*	Do the different items in one KAND group relate to one another?	Cronbach alpha ($\:\alpha\:$)			x
*Convergent validity*	Do KAND group scores correlate with other validated scales meant to capture similar behavioral constructs?	Spearman correlations (ρ)			x

*P1* phase 1, *P2* phase 2,* P3* phase 3

#### Phase 1

First, the recordings of the one-on-one semi-structured interviews were transcribed verbatim. Next, qualitative thematic data analysis [31] was performed manually via the program NVivo [32] by KT with support of SV. Each transcript and all the field notes were read several times. We subsequently reviewed the data (i.e., transcript fragments and accompanying field notes) by applying a codebook. The codebook was developed primarily deductively as interview guide topics were used to set up the original codebook. The structure was iteratively refined by adding newly collected information during data analysis. In this way, a continuous adaptation of the codebook took place as more information was collected and explored. All text fragments meaningful for the research question were labeled and sorted according to this structure of codes. Data were then reviewed and collated into themes based on their common content. Finally, these qualitative findings were compared to the TAND Checklist items to identify similarities and differences in content. As a result, a preliminary draft of the KAND Checklist was created, providing evidence to support its face and content validity.

#### Phase 2

The quantitative and qualitative data from the feedback form were analyzed and used to review face validity, content validity, and response process validity of the preliminary version of the KAND Checklist. This process involved several key steps. First, mean scores were calculated from the quantitative data. Second, qualitative feedback was thematically analyzed—using the same approach as in phase 1—to identify recurring themes. Based on this analysis, a topic list was created to guide the focus group discussion. The topic list is available upon request. Third, the focus group session was transcribed verbatim and analyzed thematically alongside field notes. Finally, the themes emerging from both the feedback forms and the focus group were compared and thoroughly reviewed to inform revisions to the KAND Checklist.

#### Phase 3

The KAND Checklist was validated using both quantitative and qualitative data collected from five questionnaires: the KAND Checklist, SRS, BRIEF, ASEBA, and a feedback form.

Quantitative analysis began with scoring the validated measures according to standard procedures. From the SRS and BRIEF, total scores were derived. From the ASEBA, the total problems, internalizing problems, externalizing problems, attention problems, and AD/H (Attention Deficit/Hyperactivity) problems scores were used. For the KAND Checklist, selected items were grouped into seven KAND groups (see Supplementary Table 2, Additional file 1), which aligned conceptually with domains covered by the validated measures. The checklist includes open-ended questions and items with two response formats: binary (yes/no) for the presence of a problem and severity ratings on a 0–10 scale reflecting the past month. KAND group scores were computed for each individual by summing binary items (scored “no” = 0, “yes” = 1), with adjustments to ensure only current difficulties were captured: if an item was answered “yes” but rated 0 in severity, it was recoded as “yes = 0.” We hypothesized that KAND groups would correlate with corresponding validated measures.

Exploratory psychometric testing was conducted to evaluate the reliability and validity of the KAND Checklist. Internal consistency was assessed using Cronbach’s alpha for each of the seven KAND groups, with values above 0.70 considered acceptable and above 0.80 considered good. Convergent validity was examined by calculating Spearman’s rho correlations between KAND group scores and the corresponding subscale scores from the SRS, BRIEF, and ASEBA. Correlation coefficients between 0.40 and 0.69 were interpreted as moderate, and those above 0.70 as strong [33]. Quantitative analyses were conducted using IBM SPSS Statistics Version 29.0.2.0 [34]. Pairwise deletion was used to handle missing data and preserve as much usable information as possible for each analysis.

In parallel, qualitative feedback from participants was used to examine face validity, content validity, and response process validity of the KAND Checklist. This feedback came from open-ended responses in the feedback form and was analyzed similarly to phase 2. Quantitative ratings from the feedback form were summarized using mean scores, providing insight into the perceived clarity, relevance, and usefulness of checklist items. Descriptive statistics were used to analyze supplementary data from the KAND Checklist (see Supplementary data, Additional file 1). Feedback data was thoroughly reviewed and used to develop and refine the KAND Checklist into the final version.

### Translation

The KAND Checklist was developed in Dutch. As this study aims to create a shared language for KAND and the widespread use of the KAND Checklist, the next step included translation to English.

A forward and back translation methodology was used. First, the Dutch version was translated into English by an individual familiar with KS and fluent in both English and Dutch. Second, feedback on the translation was collected from one individual familiar with KS and two native English speakers. Based on their feedback, adjustments were made. Third, the pre-final English version was back translated by a native Dutch speaker with good English proficiency familiar with KS, but not familiar with the KAND Checklist. Next, discrepancies between the original Dutch version and the back translated Dutch version were reviewed. The final English version was adjusted if the meaning of words/sentences conflicted with the original meaning. Finally, the final English version was formatted according to the design of the KAND Checklist.

### Researcher reflexivity

No relationship between participants and researchers was established prior to the start of the study. Participants were informed about the aims of the research, and the researchers were transparent about their positions and objectives. The researchers were new to the topic of Klinefelter syndrome. The interviews were conducted by KT (MD, researcher, female), and the focus group moderated by KT and SV (PhD, post-doctoral qualitative researcher and genetic counselor, female).

## Results

### Study participants

Participant characteristics are summarized in Tables 3 and 4.

**Table 3:** Overview number of participants per phase

	Phase 1	Phase 2	Phase 3
Individuals with KS	9	-	18
Parents of individuals with KS	11	24	29
Expert professionals	-	13	-
*Total participants per phase*	20	37	47

**Table 4:** Age distribution of individuals with KS & of children with KS reported on by parents

Age Group (Years)	Phase 1		Phase 2	Phase 3	
	Self-report	Reported by parents	Reported by parents	Self-report	Reported by parents
0–5	-	-	1 (4%)	-	1 (4%)
6–11	-	1 (14%)	4 (17%)	-	4 (14%)
12–17	-	3 (43%)	4 (17%)	-	7 (24%)
18–25	4 (45%)	3 (43%)	8 (33%)	1 (5.5%)	7 (24%)
26–44	3 (33%)	-	6 (25%)	11 (61%)	7 (24%)
45–64	2 (22%)	-	1 (4%)	5 (28%)	3 (10%)
65+	-	-	-	1 (5.5%)	-

In phase 1, sixteen interviews with nine individuals with KS and eleven parents of individuals with KS were conducted (*N* = 20). These consisted of seven interviews with an adult individual with KS, two interviews with an adult individual with KS and one of their parents, two interviews with two parents of an individual with KS, and five interviews with one parent of an individual with KS. Phase 2 involved twenty-four parents and thirteen expert professionals in (neuro)psychology, neurodevelopment, pediatric endocrinology, genetics, fertility, gynecology, speech therapy, pediatric physiotherapy, and social nursing (*N* = 37). Four professionals withdrew after signing the informed consent form because of time constraints or because they felt the topic was outside of their expertise. Five parents participated in the focus group. In phase 3, eighteen individuals with KS and twenty-nine parents participated (*N* = 47). Two parents were excluded after participation because their child had a 48,XXXY karyotype instead of 47,XXY. Two individuals with KS participated in phase 1 and 3. Thirteen of the twenty-nine parents participated in both phase 2 and phase 3.

### Phase 1 results

#### From TAND to KAND-PL: the addition of Klinefelter specific themes

Below, we outline the key adjustments and additions made to the TAND Checklist based on themes identified through qualitative analysis. These insights guided the development of the preliminary KAND Checklist. A full overview of all adjustments made from the TAND Checklist to the preliminary version of the KAND Checklist can be found in supplementary data.

Like the TAND checklist, the KAND-PL addresses developmental milestones, current level of functioning, overall impact score, additional concerns, individual priorities, coping strategies, and personal strengths/qualities. It follows the same overarching structure consisting of six main domains: difficulties with behavior, psychiatric disorders, intellectual ability, academic skills, neuropsychological abilities, and psychosocial functioning. In total, the KAND-PL consists of 13 domains, an introduction, and a new background information section.

##### Difficulties with behavior

All items from the TAND Checklist were retained. In addition, new KAND items were created to address verbal and non-verbal communication difficulties, pragmatic language use, speaking difficulties, age-inadequate behavior, and an open-ended item to mention other behavioral difficulties. Participants described mood instability and the impact of testosterone treatment on emotional regulation: *“Mood swings happen in parallel with the testosterone treatment. The treatment is performed every three weeks*,* and there is a clear difference every week. Let’s say*,* the first week*,* I feel euphoric. The second*,* somewhere in between*,* and the last week*,* I am feeling a lot less energetic*,* more tired.”* – adult with KS (19 y). Similarly, difficulties in emotion regulation and anger outbursts were highlighted: *“Yes*,* it happens*,* because he cannot always express that he is angry*,* and then the outbursts can be excessive.”* – parent (child with KS, 12 y).

Challenges in social communication were also frequently mentioned, with individuals with KS struggling to understand/define literal and figurative language: *“Like*,* for example*,* when they say: ‘You have done great.’ I do not know if they meant it sarcastically or not. Sometimes I do not fully understand what others mean.”* – adult with KS (20 y). Pragmatic language difficulties also contribute to frustration and misunderstandings: *“No matter what*,* communication is hard for him*,* also at school. And it becomes more difficult with age*,* because everything is more nuanced. He often gets the feeling that they do not understand him. He says: ‘I just cannot explain’ or ‘they don’t know what I mean.’”* – parent (11 y). Similarly, word-finding difficulties were frequently reported as barriers to communication, sometimes leading to behavioral challenges: *“Sometimes he can be aggressive. When he was little*,* it was because he couldn’t find the right words to explain. Therefore*,* when he got mad*,* instead of yelling or speaking*,* he rather knocked or kicked.”* – parent (17 y).

##### Academic skills

In addition to the TAND Checklist items, new KAND items on the presence or absence of learning disorders and the level of education were introduced.

##### Psychosocial functioning

The domain of psychosocial functioning differs substantially from the TAND Checklist. It is composed of four subdomains:

(1)* Social skills and relationships* – This subdomain consists of items concerning difficulties with social interaction, contact with peers and adults, and relationships (friendships and romantic relationships). Social difficulties such as bullying, social exclusion, or withdrawal are also addressed. Parents and individuals with KS described how language difficulties impact their ability to interact with adults: *“When someone comes to visit*,* he will run away. I think it is because he has difficulties explaining things. He is shy and introverted*,* but I know it is because of language difficulties*,* especially with adults.”* – parent (18 y). Delayed puberty and differences in development were also mentioned as affecting peer relationships: *“The difference at some point was that my peers had already entered puberty*,* and I didn’t. This has sometimes resulted in conflicts. At the moment*,* there is no problem as far as I know.”* – adult with KS (61 y).

(2)* Self-perception and mental health* – Items were added on low self-confidence, concerns about mental health, negative feelings due to KS, compulsive/intrusive thoughts, and thoughts about self-harm or suicide. Some individuals with KS talked about depressive symptoms and struggled with self-image: *“In my youth, I used to suffer from KS a lot. Everybody was happy, and for me, everything was black.”* – adult with KS (42 y). Moreover, some individuals reported positive self-perceptions despite challenges: *“I am proud of what I achieved, and I think I am a beautiful and kind person. My self-image is not as good as I would like, but at the moment, I know who I am and what I can.”* – adult with KS (20 y).

(3)* Problems in different life contexts (family and work)* – This subdomain includes items on family stress, the impact of KS on professional life, and the need for additional support. Parents emphasized the adaptability required in family life: *“We adapted from the moment he started to have difficulties, not from the moment of diagnosis.” … “I cannot work full-time, for example.”* – parent (12 y). Parents shared their greatest burden being: *“The difficulty with KS is when you look at those children. They grow up pretty normal and you do not see anything about them. Therefore, normal behavior is expected (but this is not always the case in reality).”* – parent (18 y).

(4)* Body perception and fertility* – This section explores concerns about body image, physical difficulties, and fertility. Participants expressed grief over infertility: *“About fertility, I regret being infertile. Like I missed out on something, because having your own child is still different.”* – adult with KS (40 y).

##### Background information

A new background section was added, focusing on the journey of diagnosis and access to care. Participants described their experiences with the timing and communication of diagnosis: *“When I was diagnosed*,* my parents did not inform me*,* because they also heard about infertility problems. They thought that if we tell him now*,* it will go wrong. Therefore*,* they only told me when I was 24 years old.”* – adult with KS (42 y). Others described the diagnosis as a turning point for understanding their condition: *“The diagnosis is like a key*,* it opens gates. If you do not have the key*,* you don’t know what is different. However*,* if you do*,* it gives you context. The realization that you are not alone.”* – adult with KS (42 y). Finally, concerns were raised about the continuity of care: *“Our care trajectory was quite difficult at the beginning. We did not know what the intentions were. First*,* he was treated by doctor X*,* then suddenly he was treated by another doctor and then again by another doctor. Currently*,* he is being treated by the same doctor all the time. It goes truly well. Still*,* sometimes all of a sudden*,* we get a letter that we need to see another health care provider*,* and we do not know who we will meet. This is something on which more information should be given to parents and patients.”* – parent (18 y).

### Phase 2 results

#### Quantitative feedback – feedback form

**Fig. 2:**
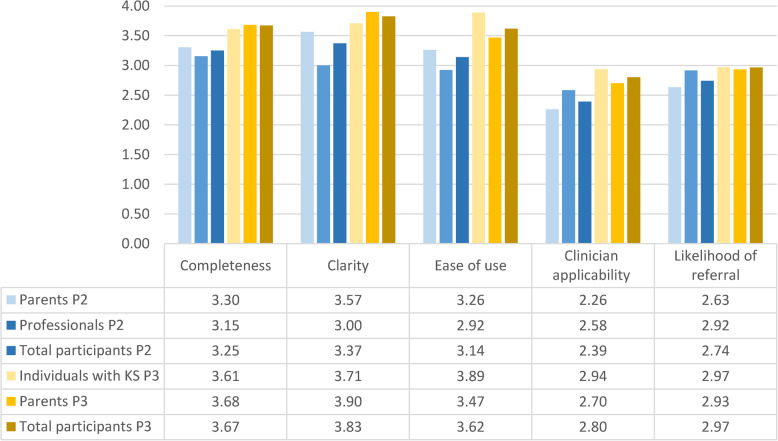
Quantitative feedback phase 2 (*n* = 36) and phase 3 (*n* = 47). Visualizes the mean scores from the feedback form per item, participant group, and phase. P2 = phase 2, P3 = phase 3

An overview of the quantitative data from the feedback form in phase 2 can be found in Fig. 2. Expert professionals gave the highest rating on completeness (mean score (x̄) = 3.15) and lowest on clinician applicability (x̄=2.58). Parents gave the highest rating on clarity (x̄=3.57) and lowest on clinician applicability (x̄=2.26). In total, participants gave the highest rating on clarity (x̄=3.37). Parents (x̄=3.57) gave a higher rating on clarity than expert professionals did (x̄=3.00). The second highest score was obtained for completeness (x̄=3.25) with parents (x̄=3.30) giving a higher rating than expert professionals (x̄ =3.15).

#### Qualitative feedback – feedback form and focus group

All participants (*n* = 37) provided written feedback on the KAND Checklist, which was used in qualitative analysis across the five topics of the feedback form. This feedback offered valuable insights for improving the checklist. From the analysis of the qualitative data from both the feedback form and the focus group, seven overarching themes for potential enhancement emerged. These themes are discussed in detail below.

##### Clarity

Participants emphasized the need for clearer wording and additional examples to improve comprehension. Some found technical jargon challenging, suggesting that simplifying and rephrasing certain items would increase accessibility—particularly for individuals with KS, who may experience language difficulties. The domain of current level of functioning and psychosocial functioning in particular required clarification. Additionally, participants found it unclear whether certain questions referred to the individual with KS or the caregiver completing the checklist.

##### Content - KAND-specific items

Regarding the completeness of the KAND Checklist, several suggestions were made to elaborate existing domains and/or add more items/domains. For example, participants proposed deepening the background information section and adding more detailed questions. Others suggested including more specific items on neuropsychological functioning, current level of functioning, and social skills. Missing items, such as difficulties with social cognition, challenges due to lack of self-insight, and tics/compulsive acts, were also mentioned. The need to add items on ‘addiction’ and ‘self-sufficiency/independence’ was raised most frequently.

The focus group participants viewed the extensiveness of the KAND Checklist as a valuable resource for gathering information and guiding individuals toward appropriate support. However, they also expressed concerns that the length and depth of the checklist might be overwhelming or confronting for individuals with KS. Adding to the results of the feedback form, participants of the focus group also suggested including more questions on adult situations and parental needs.

##### Layout and structure

Some participants suggested rearranging the sequence of questions by starting with lighter or positive topics and ending with challenging topics. Others felt that it was beneficial to conclude with positive aspects. Additional suggestions included providing more writing space and improving the visual layout of the checklist.

##### Target group

The target group for the KAND Checklist includes individuals with KS, their families, and healthcare providers. However, some participants felt that this group was too broad. They expressed a need for an age-specific version of the checklist such as separate versions for adults and children, as well as a multiperspective checklist tailored to individuals with KS, their families and healthcare providers. Other participants raised concerns that the checklist in general might not be suitable for the target group. They feared that the content was too difficult, overwhelming, or extensive for the population of interest.

##### Aims and outcome

Several focus group participants suggested adding a section with the defined purpose and expected outcome of the checklist in the introduction, as this information was absent or unclear according to them. A few participants preferred a different objective for the checklist, in the sense that a more personal description of one’s own situation over the years should be included. In terms of outcome, the KAND Checklist was considered ‘a good starting point’ for prevention or additional care. Others felt that at the end of the checklist, information about further steps or referrals should already be provided. They had the general feeling that support or follow-up for KS is limited in society.

##### Positive feedback

Overall, the KAND Checklist was well-received and appreciated. Participants gladly shared their experiences, and some said that they gained insights into KAND difficulties from taking part in the study. The focus group members acknowledged that the preliminary version was just a starting point, noting that improvement will take time. They also highlighted that, in terms of outcomes, healthcare providers need time to learn about the checklist and ways of using it. Nevertheless, it was clear that the checklist offers significant added value for both healthcare providers and parents.

##### Future considerations

During the focus group, parents suggested developing different versions of the KAND Checklist according to age (child/adult) or the person completing the checklist (individual with KS/parent) and providing an online, easy to complete KAND Checklist. In the search for care for individuals with KS in general, parents advocated introducing strategies/tools for parents and individuals with KS to cope with and discuss the diagnosis of KS. Based on their own experience, they urged health professionals to improve their knowledge of KS and the negative perceptions surrounding KS in general.

#### From KAND-PL to KAND-PF

All changes to the preliminary version of the KAND Checklist to develop the pre-final version of the KAND Checklist are organized in Table 5.

**Table 5:** Overview of changes to the KAND-PL to develop the KAND-PF

Part of KAND-PF	Description of changes
All questions	- B1 language level - More examples per item - Rewriting directions per question and item
Layout	- Page and question numbering - Separating questions by frames
Introduction	- Adding disclaimer: ‘This list does not tell you what exactly is going on or provide solutions. Always talk about it with someone who can help, such as a doctor.’
Instructions Checklist	- Clarifying the construction and goal of the two-part multiple-choice questions - Rewriting ‘Instructions Checklist’: simple, clear and concise
Background information	- ‘Diagnosis KS’: adding ‘who communicated the diagnosis and how’ - ‘Care and support’: two questions merged into one
Question 1: Developmental milestones	- Changing answer options in ‘normal’ or ‘later’
Question 2: Self-sufficiency	- Question 2 changed into ‘Self-sufficiency’ with subitems: 'Self-care', 'Finances', 'Daily living', 'Social network'
Question 3: Difficulties with behavior	- Item on pragmatic language was split in 4 separate items and difficult terms were omit - New item: 3.bb. Addiction
Question 5: Intellectual ability	- 5.b: adding multiple choice option ‘above average intelligence’
Question 6: School abilities	- Level of education changed into a multiple-choice question instead of open ended
Question 7: Neuropsychological abilities	- Technical jargon stated with examples - New item: 7.i. Social cognition
Question 8: Psychosocial functioning	Subquestions reorganized: - 8.1 Social skills and relationships - 8.2 Body perception - 8.3 Emotional well-being - 8.4 Impact of KS in different life contexts such as family and professional contexts New item: - 8.2.c. Low energy or fatigue Composite items: - 8.3.a. Low self-esteem - 8.3.c. Negative emotions because of the diagnosis of KS - 8.4.a. Impact on your family life Adding safety disclaimer with item 8.3.e. Thoughts of hurting oneself or ending your life 8.4. Impact of KS - Rephrased to focus on the respondent’s personal experience of the impact of KS, rather than the individual with KS. - “Did you (the person completing the questionnaire):”
Question 11: Priority list	- Excluding item ‘more professional care’

### Phase 3 results

#### Internal consistency - Cronbach’s alpha

All the KAND groups demonstrated good internal consistency, with Cronbach’s alpha values ranging from 0.726 to 0.885. The highest internal consistency was observed in the social communication and behavior group (α = 0.885), the executive function group (α = 0.869), and the total behavior group (α = 0.864), indicating that the items within these groups are closely related. The attention problem group (α = 0.794), internalizing behavior group (α = 0.776), and externalizing behavior group (α = 0.726) presented slightly lower, but still acceptable, internal consistency.

#### Convergent validity – Spearman’s rho

To assess the convergent validity of the KAND Checklist, it was tested alongside three frequently used validated measurements: the ASEBA, SRS, and BRIEF. This was done to examine how well the content of the KAND Checklist aligns with established measures. Correlations between the KAND Checklist groups’ scores and the corresponding scales from the validated measures were calculated, as presented in Table 6.

**Table 6:** Spearman’s Rho correlations between KAND groups and validated behavioral measures

KAND groups	Validated behavioral measures	Spearman’s rho
Total Behavior KAND group	Total ASEBA	**0.719**
Internalizing KAND group	Internalizing ASEBA	*0.634*
Externalizing KAND group	Externalizing ASEBA	**0.730**
Attention KAND group	Attention ASEBA	*0.579*
	AD/H problems ASEBA	*0.618*
Social behavior KAND group	Total SRS	**0.799**
Executive functioning KAND group	Total BRIEF	*0.662*

Spearman’s rho values are presented for the correlation between KAND groups and validated behavioral measures. Bold values indicate strong correlations (ρ ≥ 0.70); *italicized* values indicate moderate correlations (*ρ* = 0.40–0.69). *ASEBA* Achenbach System of Empirically Based Assessment, *SRS* Social Responsiveness Scale, *BRIEF* Behavior Rating Inventory of Executive Function, *AD/H* Attention Deficit/Hyperactivity

The results demonstrate moderate (Spearman’s *ρ* = 0,40 − 0,69) to strong (*ρ* = 0,70 − 0,89) correlations. The highest correlation is found between the social behavior group and the total SRS score (*ρ* = 0.799). The total behavior and externalizing KAND groups were strongly correlated with, respectively, the total ASEBA score (*ρ* = 0.719) and externalizing (*ρ* = 0.730) ASEBA scores, whereas the internalizing KAND group was moderately correlated with the internalizing ASEBA scores (ρ = 0.634). Furthermore, the attention group correlated moderately with the attention ASEBA scores (*ρ* = 0.579) and AD/H problems ASEBA scores (*ρ* = 0.618), and the executive functioning group correlated moderately with the total BRIEF score (*ρ* = 0.662).

#### Quantitative feedback – feedback form

Overall, participants rated the KAND Checklist higher in phase 3 than in phase 2 (Fig. 2). The highest-rated items were clarity (mean score (x̄) = 3.83) and completeness (x̄ = 3.67). Individuals with KS particularly highly rated ease of use (x̄ = 3.89), whereas parents gave the highest rating to clarity (x̄ = 3.90). However, both parents (x̄ = 2.94) and individuals with KS (x̄ = 2.70) gave their lowest scores—indicating a neutral to good rating—to the likelihood of others using the KAND Checklist.

#### Qualitative feedback – feedback form

Out of the 47 participants, 35 provided both written feedback and item scores, whereas 12 scored only feedback items. All the feedback can be grouped into three categories: improvements, positive feedback, and next steps.

##### Improvements

A few participants made minor suggestions to increase the ease of use, clarity, layout, and completeness of the KAND Checklist. Some struggled with the two-part multiple-choice questions and reported that certain items were not yet applicable due to the age of the individual with KS. The final adjustments made to the KAND Checklist are detailed in Table 7. These include clarification of instructions, expansion of the background information section, and improvements to the layout.

**Table 7:** Overview of changes to KAND-PF to develop KAND 2025

Part of KAND 2025	Description of changes
Instructions checklist	- Clarifying aim of the questions - Clarifying perspective of the questions - Clarifying introduction: N/A = no
Background information	- ‘Diagnosis KS’: clarifying question and adding item on support related to the diagnosis - ‘Care and support’: adding example on medical care/medication
Layout	- More space for notes - Layout more tight/clear - Changing order of domains

KAND 2025 = KAND Checklist (Final version 2025)

##### Positive feedback

Many participants shared positive feedback on various aspects of the KAND study. Some noticed a significant improvement in the preliminary version of the checklist and praised its completeness. Others highlighted the importance of the KAND Checklist for individuals with KS and their families, noting how it improves visibility and empowers them to look for appropriate assistance. Additionally, some participants expressed gratitude for valuing parents’ perspectives, for the improved understanding of KS and KAND, and for how the checklist can help individuals recognize their own experiences in the descriptions - fostering self-recognition.

##### Next steps

Several participants hoped that the KAND Checklist would be widely adopted by healthcare providers working with individuals with KS. The tool could help them gain (more) insight into KS-specific difficulties and provide effective personalized support. Some participants envisioned next steps, such as establishing a support group for individuals with KS to share experiences.

#### From KAND-PF to KAND 2025

Following the validity testing, the checklist was further refined based on feedback collected through the feedback form. The subsequent adjustments can be found in Table 7. This result became the final KAND Checklist, which was then translated via a forward-backward procedure. The full version of the checklist is provided in Additional file 2 and an overview of the structure can be found in Table 8.

**Table 8:** Structure of the KAND Checklist (Final version 2025)

Section/domain – Content
**Background information:** Diagnosis KS & care and support
**Question 1 :** Developmental milestones
**Question 2 :** Difficulties with behavior
**Question 3 :** Intellectual ability
**Question 4 :** Neuropsychological abilities
**Question 5 :** Psychiatric disorders
**Question 6 :** Academic skills
**Question 7 :** Self-sufficiency
**Question 8 :** Psychosocial functioning 8.1 Social skills and relationships 8.2 Body perception 8.3 Emotional well-being 8.4 Impact of KS in different life contexts such as family and professional contexts
**Question 9 :** Assessment of the impact of KS
**Question 10 :** Additional concerns
**Question 11 :** Prioritizing list
**Question 12 :** Strategies
**Question 13 :** Strengths and positive traits

## Discussion

Using a participatory research approach, we have developed, initially validated, and translated the KAND Checklist, a tool to screen for neurodevelopmental difficulties associated with Klinefelter syndrome.

### From TAND to KAND

Key modifications included adding a background information section, inserting more detailed items/questions on language and communication, difficulties with behavior, and reorganizing and elaborating the psychosocial functioning items. These modifications are discussed below and substantiated by literature and experiences of participants.

The participants in this study experienced several barriers during their diagnostic process, including issues with the timing and communication of the diagnosis, as well as inadequate support. Studies show that families, regardless of whether the diagnosis is given in the prenatal or postnatal period, often feel unsettled by healthcare providers’ limited familiarity with the condition of KS and its implications. This lack of awareness and knowledge significantly impacts medical management, leaving individuals with KS feeling unsupported and uninformed [18, 35]. A recently developed patient education program for young people with differences in sex development (DSD) and their parents has demonstrated success in improving diagnosis-specific knowledge and empowerment [36]. Further implementation of this program could address the urgent need for reliable information and enhanced communication in healthcare settings for conditions like KS [18, 37, 38]. The KAND Checklist responds to this need by incorporating a background information section to guide healthcare providers in understanding an individual’s diagnostic journey, evaluating their current support systems, and fostering appropriate family guidance.

Most individuals with KS experienced language and communication difficulties with multiple parents consulting a speech therapist at some point. This finding is consistent with previous research reporting a high prevalence of language, literacy and social-pragmatic deficits in individuals with KS [12]. Parents expressed their concerns about the impact of these difficulties on their child’s life, causing feelings of frustration, anger outbursts and obstacles to social interactions. As early detection and intervention may reduce the risk for academic, behavioral and psychosocial challenges later in life, addressing these concerns is important [12, 18]. Therefore, items on verbal and non-verbal communication, pragmatics, and speaking were added to the KAND Checklist.

Compared with the TAND Checklist, the psychosocial functioning domain of the KAND Checklist has been extensively revised to provide a deeper understanding and discussion of psychological and social difficulties in KS. This domain highlights underexplored topics in research in individuals with KS such as mental health, sexuality, and the impact of KS on family dynamics and careers [18, 39]. Furthermore, it includes high-impact characteristics such as physical issues, fertility problems, and social communication difficulties [18]. As these issues are often difficult to detect and discuss, this domain gives individuals the opportunity to articulate their own experiences and difficulties based on recognition without having to put them into words themselves. The checklist can be the first step in the chain of identification, acknowledgment, and management of psychosocial difficulties, ultimately improving a person’s wellbeing and quality of life.

### Validation and translation

By systematically gathering qualitative and quantitative data contributing to scientific validity, we developed a checklist that is both reliable and meaningful to the target population. Across all study phases, input from individuals with KS, parents, and healthcare professionals provided evidence for face, content, and response process validity. The iterative feedback confirmed that the checklist captures the most relevant KAND-related difficulties, uses clear and accessible language, and is feasible for both self- and proxy-completion. These findings demonstrate that the KAND Checklist reflects real-world experiences and needs, and that its structure and wording support practical use. Adjustments included adding items on addiction and fatigue, changing the ‘level of functioning’ to measures of self-sufficiency, and simplifying the language to remove technical jargon. These refinements underscore the importance of adapting tools such as the KAND Checklist to the neurocognitive and linguistic needs of individuals with KS, as supported by their difficulties with language and executive functioning [4, 9, 12].

The quantitative findings further reinforced the checklist’s qualities. The participants’ ratings of the checklist’s completeness improved significantly from phase 2 to phase 3, confirming its ability to comprehensively measure relevant Klinefelter-associated challenges. Internal consistency, as measured by Cronbach’s alpha within conceptually related item groups, provides preliminary evidence that the checklist items are related and likely measure a cohesive construct. However, based on the current findings, no statements can be made regarding the interrelatedness of all items and the possible influence of external factors on the construct, highlighting the need for future exploratory factor analyses to further investigate the underlying structure of the checklist and its components [40]. Convergent validity, assessed by calculating Spearman’s rho correlations with other behavioral measures, offers initial support that item groups within the KAND Checklist reflects constructs similar to those captured by existing measures, contributing to its relevance and applicability for clinical and research purposes.

While the KAND Checklist successfully assembles the most relevant difficulties associated with KS and pointed out by lived experts, the findings do not indicate the presence of a distinct ‘KAND profile’. Instead, the results highlight the heterogeneity of KS-associated challenges with some, such as language and executive function impairments, being more frequently reported than others, such as psychiatric vulnerabilities or addiction. This variability underscores the importance of using the KAND Checklist as a flexible tool to identify individual needs, rather than as a diagnostic framework for a singular KS phenotype.

As the KAND Checklist aims to improve the understanding of and communication about KAND, it is essential to present individuals with KS with a version of the KAND Checklist that they comprehend. The forward-backward translation process highlighted the importance of carefulness and attention to preserve the clarity and comprehensibility of the original KAND Checklist. The multiple perspective review of the forward translation provided valuable insights and nuances and allowed a substantiated translation.

### Usability

In terms of usability, all instructions for the KAND Checklist are provided on the first page, requiring no prior training. We developed a pen-and-paper version to be completed at least annually, either on one’s own initiative or at the request of a healthcare provider. Checklist results should be discussed with a healthcare provider, as the tool does not exactly tell you what is going on or provide solutions. The background section is important at first use and should be updated if information changes or new challenges arise. The KAND Checklist is intended for individuals of all ages. Some items may not be or not yet be applicable, in which case instructions indicate to answer “no”.

### In what ways can the findings from this work benefit other (rare) diseases?

In recent years, similar screening tools have been developed to assess neurodevelopmental difficulties associated with TSC, Duchenne muscular dystrophy (DMD), and Dravet syndrome (DS). These projects were developed for similar reasons: awareness, detection, and management of disease-specific nonphysical challenges are not part of common healthcare practices. Likewise, these complex conditions would benefit from holistic, patient-centered and multidisciplinary approaches. Although the methodology in these initiatives is similar, they all address different challenges and problems inherent to the conditions in question. From these projects, we learn that key factors in the development of disease-specific neurodevelopmental initiatives are input and contribution of lived and professional experts to ensure that outcomes are relevant and valuable for all those involved. We learn that, especially in underdiagnosed and rare diseases, access to reliable information and training for healthcare professionals is fundamental to improving care. Further, we see that these initiatives begin by establishing a shared language to identify and discuss these often underassessed difficulties. Finally, the development of these tools depends on iterative refinement through multiple phases of research. However, the feasibility and inherent limitations of disease-specific measurements – especially in rare diseases - must be considered. Notably, caregiver’s concerns often differ from clinical evaluation. The greater need within the community lies more in practical tools and strategies to manage these challenges in daily life, underscoring the importance of continued research and development [25, 27, 41–43].

### Strengths and weaknesses

One of the greatest strengths of this study is its participatory nature. Active involvement of families and individuals with KS ensured the checklist reflects lived experiences and strengthened its content validity, clarity, face validity, and practical relevance. This process also empowered participants and increased the likelihood that the checklist would be meaningful and likely to be implemented in clinical and community settings, reflecting the core principles of participatory research described by Abma et al. [44]. Although the level of participation varied across study phases, the iterative collaboration between researchers and the KS community resulted in a checklist that integrates scientific evidence with experiential knowledge.

A major strength of this study is the continuous validation process, which ensured that the checklist evolved into a well-adapted tool for individuals with KS, their families, and healthcare professionals. By integrating scientific literature, expert opinions, and participant feedback, the checklist was refined to address previously underrepresented difficulties and was adjusted for linguistic clarity and accessibility. This process enabled the introduction of a shared language for discussing KS-associated difficulties. However, while the checklist successfully compiles relevant KAND-related challenges, has clear checklist instructions, and requires no education prior, it does not offer direct solutions or deliver first step advice after completion. Further research should focus on how to guide and aid individuals and professionals based on checklist outcomes.

Another limitation, also raised by participants, is that although the checklist was designed for individuals with KS of all ages and their parents, some items (e.g., those related to employment or development) may not be directly applicable to all age groups. This feedback was acknowledged but addressed only to a limited extent. Additionally, we have limited data for the youngest age range: no individuals with KS younger than 18 years were included, and only a few parents of younger children participated. Developing age-specific versions and testing them in younger populations (young children and adolescents) may improve usability and support early identification of challenges.

Additionally, cultural generalizability remains a limitation as the checklist requires adaptation to be applied in a culture- or country-specific setting. Availability in multiple languages and versions adjusted to the language level of the person with KS will be one of the next steps to maximize its global adoption. No caregivers participated across the study phases, highlighting the need for future research to actively engage this user group.

Finally, owing to the limited sample size, advanced psychometric analyses such as exploratory factor analysis could not yet be conducted. Although the internal consistency results indicate that the checklist items capture related constructs, larger and more diverse samples are needed to establish structural validity and to further explore potential KAND clusters. Applying the COSMIN checklist in future studies could strengthen the validation process, particularly if the KAND Checklist is to be further developed and evaluated as a patient-reported outcome measure [45]. 

### Future

Implementation of the KAND Checklist in clinical practice is the next logical step. A way to enhance visibility and accessibility is dissemination of the tool through expert networks, translation into multiple languages, and an online version alongside the paper format. In addition, accurate information on KAND, as well as the checklist itself, should be readily available, misconceptions should be addressed, and efforts should be made to improve healthcare providers’ knowledge of KAND.

The results of this study underscore the importance of adopting a holistic approach to KS care, emphasizing multidisciplinary collaboration to provide comprehensive and effective support for individuals with KS. Current guidelines, such as the European Academy of Andrology Guidelines for KS (2020), offer limited insight into managing KAND. While their recommendation for early monitoring of psychosocial challenges is a critical step, it is not sufficient on its own [46]. To ensure long-term international integration, broader structural collaboration will be essential. An international consortium focused on KAND could help define research priorities, coordinate further validation in larger and international cohorts, and support global implementation. Involving the international KS community in these efforts will ensure alignment with the lived experiences and evolving needs of individuals with KS and their families. The consortium could serve as a platform to sustain participatory research, guide tool development, and facilitate the creation of international KAND guidelines.

While establishing these guidelines and fostering international collaboration are important next steps, their true impact depends on effective implementation and coordinated multidisciplinary action in clinical practice. Identifying KAND should not end with referral to a specialist but should prompt integrated action across healthcare, education, and support systems [39]. In parallel, new tools and strategies should be explored through participatory research to align with the priorities of individuals with KS and their families. Promising initiatives, such as social management training, which aims to enhance individuals’ ability to regulate their thoughts, emotions, and behaviors, should be further explored and tested. A multi-stakeholder priority-setting exercise could be a way to decide on next steps together with the community [47, 48].

## Conclusion

This study developed the KAND Checklist to systematically identify and address neurodevelopmental difficulties in individuals with KS. Adapted from the TAND Checklist, key modifications were made to better reflect the needs of individuals with KS and their families, including expanded domains on language, communication, and psychosocial difficulties. Efforts were also made to enhance usability by simplifying language, reducing technical terms, and adding examples for clarity.

The validation process demonstrated the checklist’s reliability and relevance. While it does not define a distinct “KAND profile” due to the heterogeneity of KS-associated challenges, it provides a structured framework for recognizing and discussing these difficulties. The checklist is intended to facilitate conversations between healthcare providers, individuals with KS, and their families, potentially improving awareness and support.

Future efforts should focus on the checklist’s implementation by improving its accessibility through translation and digital formats and integrating it into existing care pathways. Continued collaboration with the community to develop complementary tools for managing KAND, alongside identification, is a priority for improving care and support for individuals with KS.

## Supplementary Information

Additional File 1. Supplementary data.

Additional File 2. KAND Checklist.

## Data Availability

The datasets generated and/or analyzed during the current study are available from the corresponding author on reasonable request.

## Abbreviations

ABCL: Adult Behavioral Checklist
AD/H: Attention Deficit/Hyperactivity
ASEBA: Achenbach System of Empirically Based Assessment
ASR: Adult Self Report
BRIEF: Behavior Rating Inventory of Executive Function
CBCL: Child Behavioral Checklist
DMD: Duchenne muscular dystrophy
DS: Dravet syndrome
EN: English
IK: Individuals with Klinefelter syndrome
KAND: Klinefelter-Associated Neurodevelopmental Difficulties
KAND 2025: Klinefelter-Associated Neurodevelopmental Difficulties Checklist (Final version 2025)
KAND-PL: Klinefelter-Associated Neurodevelopmental Difficulties Checklist – Preliminary version
KAND-PF: Klinefelter-Associated Neurodevelopmental Difficulties Checklist – Prefinal version
KS: Klinefelter syndrome
NL: Dutch
P1, P2, P3: Phase 1, Phase 2, Phase 3
SRS: Social Responsiveness Scale
TAND: TSC-associated Neuropsychiatric Disorders
TSC: Tuberous sclerosis complex
y: Years
