# Supplementary material for: Development and validation of the Klinefelter-Associated Neurodevelopmental Difficulties (KAND) Checklist: a three-phase mixed-methods study
Source: J Neurodev Disord. 2026 Jan 30;18:7. doi: 10.1186/s11689-025-09670-0 (PMC12879426; doi:10.1186/s11689-025-09670-0)
Supplement: Supplementary file 1 — Additional File 1. Supplementary data. [file 11689_2025_9670_MOESM1_ESM.docx]

# Supplementary data

#### **PHASE 1 – RESULTS**

**Supplementary Table 1: Overview of all adjustments from the TAND Checklist to the preliminary version of KAND Checklist**

| **KAND-PF** |
| --- |
| **Background information** |
| - **Journey of Diagnoses: timing + communication** - **Care and support** |
| Basic Developmental milestones |
| Current level of functioning |
| - ~~Mobility~~ |
| Behavioral concerns |
| - **Difficulties with verbal communication** - **Difficulties with non-verbal communication** - **Difficulties with pragmatic language**   - **Difficulties adapting one's own language to a given social situation**   - **Difficulties with distinguishing literal and figurative language**   - **Difficulties communicating a message concisely and clearly**   - **Difficulties explaining emotions or feelings** - **Speaking difficulties** - **Age inadequate behavior** - **Any other behavioral difficulties** |
| Diagnosis psychiatric disorder |
| Intellectual ability |
| Academic skills   - **Level of education** - **Learning disorder** |
| Neuropsychological skills |
| Psychosocial functioning   - **Social skills and relationships**    - **Difficulties with social interaction**   - **Difficulties in contact with peers**   - **Difficulties in contact with adults**   - **Social difficulties such as bullying, social exclusion or social withdrawal**   - **Relationship difficulties**     - **Friendships**     - **Romantic relationships**   - **Concerns about social skills**   - **Concerns about social support**   - **Concerns about sexuality, such as low sexual interest or very high sexual interest or other concerns.**   - **Concerns/confusion about gender identity** - **Self-perception and mental health**    - **Low self-confidence**   - **Concerns about your mental health/well-being**   - **Frustrations or other negative feelings due to KS**   - **Negative thoughts due to KS**   - **Compulsive/intrusive thoughts (recurring, unwanted, and uncontrollable thoughts)**   - **Thoughts about hurting yourself or ending your life** - **Problems in different life contexts such as family and professional context** - **Impact on family life** - **Impact on professional life** - **Need for additional support such as career counseling or guidance** - **Body and physical functioning** - **Body image and perception difficulties (how you look at your own body)** - **Concerns about physical issues** - **Difficulties with fertility** - **Concerns on fertility** |
| Parent, caregiver, or self-rating of the impact of KS (overall impact score) |
| Additional concerns |
| Prioritizing list   - **More professional support** - **More information** - **Missing topics** |
| Coping strategies |
| Personal strengths/qualities |

This table shows all added and deleted items when comparing the TAND Checklist and the first, pre-liminary version of the KAND Checklist. Additional items are showed in **bold**, deleted items are ~~crossed out.~~

#### **PHASE 2 - RESULTS**

**Supplementary Table 2 – Quotes from focus group**

| POSITIVE FEEDBACK | “I think that's very good what you've done. To make that impetus to start with this (with this research) and deliver the perfect (check)list right away will never be possible and will never be likely. This will be a list that has to be adjusted regularly. But it is a very nice start. We can only come out stronger as parents, but also as caregivers who learn to work and grow with it.” |
| --- | --- |
| POSITIVE FEEDBACK | “We did enjoyed participating in this (this study). Because it gave us another insight into the multitude of problems and possible occurring things in such an individual (with KS). It's all a lot of.” |
| KAND-SPECIFIC ITEMS | “I think especially being addiction-prone and impressionable worries us the most in high school. This combined with fears: taking the bus alone, going somewhere alone, going to sleep is still exciting, fear that something is going to happen to someone in our family. That is what keeps us most worried.” |
| KAND-SPECIFIC ITEMS | “Poor planning skills are often seen in normal-looking people as being lazy, try harder. So I do think that is very important.” |
| FUTURE CONSIDERATIONS | “But I think that sometimes different advice is given from healthcare providers and we could work faster on that. Parents do not have all the tools, but healthcare providers, they should give a guideline of how do you deal with this, what do you advise to tell or not to tell.” |
| FUTURE CONSIDERATIONS | “I think it is important to take those things that go differently than peers to healthcare providers so they can build knowledge on this. But also so that if tools are developed for that (these differences/difficulties), help can be provided.” |
| PARENTAL NEEDS | “For myself to add to the whole thing is somewhere an addition of what do you need as a parent. I find that a lot of parents go over the top and very often the conversations are about the child.” |
| AIMS AND OUTCOME | “BUT what will the healthcare provider do with that information (a filled in KAND Checklist)?” |

#### **PHASE 3 - RESULTS**

**Supplementary Table 3: Overview of KAND Groups and associated KAND Items**

| **KAND groups – name (number of items)** | **KAND Items - number** | **KAND Items - name** |
| --- | --- | --- |
| Total behavior (22) | 3A  3B  3C  3D  3E  3F  3G  3R  3T  3U  3V  3W  3X  3Y  3BB  8.2a  8.2c  8.3a  8.3b  8.3c  8 3d  8.3e | Anxiety  Depressed mood  Severe shyness  Mood swings  Aggressive outbursts  Temper Tantrums  Self-injury  Repetitive behavior  Age-inappropriate behavior  Interaction with peers  Hyperactivity  Paying attention or concentrating  Restless behavior  Impulsive behavior  Addiction  Body image  Low energy or fatigue  Low self-esteem  Mental health  Negative emotions because of the diagnosis of KS  Compulsive thoughts  Thoughts of hurting oneself or ending your life |
| Internalizing behavior (10) | 3A  3B  3C  3G  8.2a  8.2c  8.3a  8.3b  8.3c  8.3e | Anxiety  Depressed mood  Severe shyness  Self-harm  Body image  Low energy or fatigue  Low self-esteem  Mental health  Negative emotions because of the diagnosis of KS  Thoughts of hurting oneself or ending your life |
| Externalizing behavior (6) | 3D  3e  3f  3V  3Y  3BB | Mood swings  Aggressive outbursts  Temper Tantrums  Hyperactivity  Impulsive behavior  Addiction |
| Attention problem (6) | 3V  3w  3X  3Y  7B  7E | Hyperactivity  Paying attention or concentrating  Restless behavior  Impulsive behavior  Attention  Executive skills |
| Social communication and social behavior (19) | 3h  3i  3j  3k  3l  3m  3n  3o  3p  3q  3r  3s  3t  3u  7E  7I  8.1a  8.1b  8.1c | Language development  Finding the right words  Understanding gestures or facial expressions  Adjusting language in different social situations  Literal and figurative language  Communicating a message concisely and clearly  Explaining emotions or feelings  Unclear speech  Repeating words or phrases  Poor eye contact  Repetitive behavior  Rigid or inflexible behavior  Age-inappropriate behavior  Interaction with peers  Executive skills  Social cognition  Social interactions  Social problems  Forming, maintaining or deepening relationships |
| Executive functions (13) | 7A  7B  7C  7E  7F  7H  3d  3f  3t  3s  3w  3x  3y | Memory  Attention  Performing multiple tasks at once  Executive skills  Orientation in time and space  Processing speed  Mood swings  Temper Tantrums  Age-inappropriate behavior  Rigid or inflexible behavior  Paying attention or concentrating  Restless behavior  Impulsive behavior |

**Supplementary Figure 1: Overview of all Spearman Correlations between KAND Groups, BRIEF, SRS, and ASEBA scores**

|  | SOM TOT | SOM INT | SOM EXT | SOM ATT | SOM SOC | SOM EXE | TOT ASEBA | INT | EXT | AP | AD/H_ASEBA | TOT SRS | TOT BRIEF |
| --- | --- | --- | --- | --- | --- | --- | --- | --- | --- | --- | --- | --- | --- |
| SOM TOT | 1 | 0,882 | 0,857 | 0,786 | 0,723 | 0,78 | 0,719 | 0,699 | 0,634 | 0,558 | 0,558 | 0,58 | 0,575 |
| SOM INT | 0,882 | 1 | 0,614 | 0,537 | 0,492 | 0,496 | 0,556 | 0,634 | 0,421 | 0,492 | 0,42 | 0,337 | 0,414 |
| SOM EXT | 0,857 | 0,614 | 1 | 0,794 | 0,737 | 0,783 | 0,7 | 0,6 | 0,73 | 0,508 | 0,604 | 0,648 | 0,605 |
| SOM ATT | 0,786 | 0,537 | 0,794 | 1 | 0,684 | 0,915 | 0,66 | 0,544 | 0,7 | 0,579 | 0,618 | 0,534 | 0,643 |
| SOM SOC | 0,723 | 0,492 | 0,737 | 0,684 | 1 | 0,776 | 0,609 | 0,6 | 0,593 | 0,326 | 0,414 | 0,799 | 0,545 |
| SOM EXE | 0,78 | 0,496 | 0,783 | 0,915 | 0,776 | 1 | 0,671 | 0,549 | 0,671 | 0,586 | 0,607 | 0,618 | 0,662 |
| TOT ASEBA | 0,719 | 0,556 | 0,7 | 0,66 | 0,609 | 0,671 | 1 | 0,864 | 0,84 | 0,733 | 0,789 | 0,619 | 0,791 |
| INT | 0,699 | 0,634 | 0,6 | 0,544 | 0,6 | 0,549 | 0,864 | 1 | 0,65 | 0,548 | 0,567 | 0,643 | 0,637 |
| EXT | 0,634 | 0,421 | 0,73 | 0,7 | 0,593 | 0,671 | 0,84 | 0,65 | 1 | 0,659 | 0,776 | 0,629 | 0,785 |
| AP | 0,558 | 0,492 | 0,508 | 0,579 | 0,326 | 0,586 | 0,733 | 0,548 | 0,659 | 1 | 0,872 | 0,224 | 0,823 |
| AD/H_ASEBA | 0,558 | 0,42 | 0,604 | 0,618 | 0,414 | 0,607 | 0,789 | 0,567 | 0,776 | 0,872 | 1 | 0,392 | 0,885 |
| TOT SRS | 0,58 | 0,337 | 0,648 | 0,534 | 0,799 | 0,618 | 0,619 | 0,643 | 0,629 | 0,224 | 0,392 | 1 | 0,501 |
| TOT BRIEF | 0,575 | 0,414 | 0,605 | 0,643 | 0,545 | 0,662 | 0,791 | 0,637 | 0,785 | 0,823 | 0,885 | 0,501 | 1 |

#### Internal results from the KAND-PF

**Receiving/wishing help on behavioral difficulties**

In a group of participants recruited through health care institutions and a patient care organization, it is interesting to visualize the numbers of participants who yes/no received help in the past/present and who yes/no wishes help on this moment for difficulties addressed in the KAND Checklist.

Figure 1 illustrates the distribution of participants regarding whether they have received help and/or currently wish for help with behavioral difficulties outlined in the KAND Checklist. Of the total participants, 50% have received help for their challenges and 54% indicated currently desiring additional help. Notably, 17% of participants have not yet received help but wish to receive it, while 27% reported neither having received help nor wishing help for it. A small proportion (6%) did not respond to this question.

**Supplementary Figure 2: Visual overview of the percentage of participants who receive/not receive and wish/not wish help for behavioral challenges**

**Frequency of items**

Figure 2 illustrates the frequency with which items from questions 3 to 8 of the KAND Checklist were identified as 'ever being a difficulty' by parents and individuals with KS. The most frequently recognized difficulties included 3.i: "Difficulty finding the right words," 7.b: "Difficulty with attention," and 8.2.c: "Low energy or fatigue." Other common difficulties included 3.m: "Difficulty communicating concisely" and 8.3.a: "Low self-esteem." In contrast, the least frequently reported difficulties were from question 4 (psychiatric disorders), such as obsessive-compulsive disorder, psychotic disorder, and anxiety, as well as items like 8.2.f: "Concerns about gender identity" and 3.g: "Self-harm."

**Supplementary Figure 3: Frequency of items reported as ‘YES, ever been a difficulty’**

This figure shows per item how many times it was scored as ‘yes, ever been a difficulty’ by individuals with KS or parents from individuals with KS. The items are ordered on the x-axis from most frequent being a difficulty to least frequent. The y-axis represents the number of ‘yes, ever been a difficulty’ out of a total of 48 answers.

**KAND reported diagnosis of autism spectrum disorder versus SRS scores**

Overall, 18% of participants scored in the clinical range on the SRS, with an equal distribution of 50% having a formal ASD diagnosis and 50% not having one. Additionally, 39% scored subclinical, with only 41% of this group holding a formal diagnosis.

**Figure 3** illustrates the comparison between participants who reported having a formal Autism Spectrum Disorder (ASD) diagnosis and those who did not, alongside their scores on the Social Responsiveness Scale (SRS). Notably, 9% of participants without a formal ASD diagnosis scored very high on the SRS, while 23% scored in the subclinical range despite not having a diagnosis.

**Supplementary Figure 4: Overview of percentage of participants with/without a formal ASS diagnosis and their SRS score**

Subclinical SRS = SRS score of 61-75, Clinical SRS = SRS score of 76 or more

**KAND reported diagnosis of ADHD versus AD/HD scores ASEBA**

**Figure 4** presents a comparison between participants with a formal Attention Deficit Hyperactivity Disorder (ADHD) diagnosis and those without, along with their ADHD scores on the ASEBA. 84% did not reported having a formal diagnosis although 8% of them scored very high on the ASEBA and 16% scored subclinical.

**Supplementary Figure 4: Overview of percentage of participants with/without a formal ADHD diagnosis and their AD/HD score on the ASEBA**

Subclinical ASEBA = AD/HD score of 65-69, Clinical ASEBA = AD/HD score of 70 or more
