# Supplementary material for: Development and validation of the Klinefelter-Associated Neurodevelopmental Difficulties (KAND) Checklist: a three-phase mixed-methods study
Source: J Neurodev Disord. 2026 Jan 30;18:7. doi: 10.1186/s11689-025-09670-0 (PMC12879426; doi:10.1186/s11689-025-09670-0)
Supplement: Supplementary file 2 — Additional File 2. KAND Checklist. [file 11689_2025_9670_MOESM2_ESM.pdf]

KAND Checklist

Version 2025 - English

Some individuals with **Klinefelter syndrome** (KS) experience problems with behavior, emotions, social skills, mental health or language. These problems are different for everyone. To make it easy to talk about them, we call them KAND (Klinefelter-Associated Neurodevelopmental Difficulties): **Klinefelter-related problems with behavior and thinking.** This list was created for people with KS, parents, friends and people who care for someone with KS. The KAND Checklist helps to 1) talk about KAND more easily, 2) recognize them quickly, and 3) get the right help.

This checklist does not tell you what exactly is going on or provide solutions. Always talk about it with someone who can help, such as a doctor.

Let's start

Name of the person filling out the KAND Checklist:		Name of the person with KS:	
You are (cross):	☐ a person with KS<br>☐ parent<br>☐ carer<br>someone else, namely:<br>------------------------<br>------------------------	Date of birth (dd/mm/yy):	___ / ___ / ___
		Age:	
		Personal pronouns person with KS:	☐ he/him/his<br>☐ she/her/hers<br>☐ they/them/theirs
Date you fill in the KAND:	___ / ___ / ___	Name of interviewer (if applicable):	

Checklist instructions

The KAND Checklist is designed to check whether a person with KS or their loved ones are experiencing difficulties. Because everyone experiences events and challenges differently, there are **no right or wrong answers**.

The checklist consists mainly of **multiple-choice questions**. Some questions have **dotted lines** where you may write additional information or comments. Some questions have two parts, first asking whether [you/the person with KS]* have **ever** had trouble with, e.g., anxiety. This helps healthcare providers get a quick idea of past problems. Next, you are asked to rate with a **score from 0 to 10** how much or how little you think this has been a problem over **the last month**. This score reflects **your own feeling**; this is not a precise measurement. This allows healthcare providers to know which difficulties need attention now.

The checklist covers **several topics**: diagnosis of KS, care and support, development, difficulties with behavior, intellectual ability, neuropsychological skills, psychiatric disorders, school abilities, self-sufficiency, and psychosocial functioning. Some questions may be more or less relevant to you. **Try to complete all the questions**, even if you are unsure. When a question does not apply/ does not yet apply to you or the person with KS, answer “NO”. Completing the KAND checklist takes about 20-30 minutes.

The checklist starts by collecting background information about the diagnostic process and current care and support.

**[you/the person with KS]: As a person with KS, you read this as [you]. If you are not a person with KS, read this as [the person with KS].*

Background information - *diagnosis KS*

What was the age of the person with KS at the time of diagnosis?

- ☐ Before birth
- ☐ After birth at age: _____ year _____ months

Has the diagnosis already been communicated to the person with KS? ☐ NO ☐ YES

If YES, who communicated the diagnosis to the person with KS?

- ☐ A health care provider
- ☐ A parent
- someone else, namely: ________________________

If YES, how was the diagnosis communicated to the person with KS?

- ☐ Through a conversation
- ☐ Via phone call
- ☐ Via letter or e-mail

Would you like further information/support around discussing the diagnosis? ☐ NO ☐ YES

Background information - *care and support*

What form(s) of care and support do(es) [you/the person with KS] currently receive? (e.g., psychological counseling, speech therapy, physiotherapy, medical care or medication, extra support at school or work)?

Notes:

If you **care for someone with KS**, start with **question 1**.
If you **are a person with KS** completing the questionnaire for yourself, start with **question 2**.

Question 1: Developmental milestones

How was the development of the person with KS relative to their peers? Were they about the same age as their peers or were they older than their peers when they:

a. First smiled	☐ Normal	☐ Later
b. Sat without support	☐ Normal	☐ Later
c. Walked without holding on	☐ Normal	☐ Later
d. Used single words (not “mamama”, but eg., “car”)	☐ Normal	☐ Later
e. Used two words/short phrases (eg., “eat pear”)	☐ Normal	☐ Later
f. Were toilet trained during the day	☐ Normal	☐ Later
g. Were toilet trained at night	☐ Normal	☐ Later

Notes:

Question 2: Difficulties with behavior

Did [you/the person with KS]:

a. Ever suffer from **anxiety**, such as fear of a specific situation or object — NO / YES

If YES, how much did you experience this as a problem over the last month?

Not at all 0 1 2 3 4 5 6 7 8 9 10 *Extremely*

b. Ever suffer from **depressed mood**, such as being very sad, experiencing little pleasure, or feeling empty or worthless — NO / YES

If YES, how much did you experience this as a problem over the last month?

Not at all 0 1 2 3 4 5 6 7 8 9 10 *Extremely*

c. Ever suffer from **severe shyness**, such as struggling to start a conversation, feeling uncomfortable with attention, not daring to ask questions, or avoiding eye contact — NO / YES

If YES, how much did you experience this as a problem over the last month?

Not at all 0 1 2 3 4 5 6 7 8 9 10 *Extremely*

d. Ever suffer from **mood swings**, such as rapid and unpredictable changes in emotions — NO / YES

If YES, how much did you experience this as a problem over the last month?

Not at all 0 1 2 3 4 5 6 7 8 9 10 *Extremely*

e. Ever experience **aggressive outbursts**, such as swearing, yelling, physical violence (hitting, kicking), breaking things or threatening others — NO / YES

If YES, how much did you experience this as a problem over the last month?

Not at all 0 1 2 3 4 5 6 7 8 9 10 *Extremely*

f. Ever suffer from **temper tantrums**, such as age-inappropriate anger outbursts — NO / YES

If YES, how much did you experience this as a problem over the last month?

Not at all 0 1 2 3 4 5 6 7 8 9 10 *Extremely*

g. Ever suffer from **self-harm**, such as hitting, biting, scratching oneself — NO / YES

If YES, how much did you experience this as a problem over the last month?

Not at all 0 1 2 3 4 5 6 7 8 9 10 *Extremely*

h. Ever suffer from delayed or abnormal **language development** — NO / YES

If YES, how much did you experience this as a problem over the last month?

Not at all 0 1 2 3 4 5 6 7 8 9 10 *Extremely*

i. Ever have difficulty **finding the right words** — NO / YES

If YES, how much did you experience this as a problem over the last month?

Not at all 0 1 2 3 4 5 6 7 8 9 10 *Extremely*

j. Ever have difficulty understanding and/or using **gestures or facial expressions** — NO / YES

If YES, how much did you experience this as a problem over the last month?

Not at all 0 1 2 3 4 5 6 7 8 9 10 *Extremely*

k. Ever have difficulty **adjusting language in different social situations**, such as more polite language at school or work, relaxed language with friends, or simple language with children — NO / YES

If YES, how much did you experience this as a problem over the last month?

Not at all 0 1 2 3 4 5 6 7 8 9 10 *Extremely*

Did [you/the person with KS]:

l. Ever have difficulty understanding the difference between **literal and figurative language**, such as understanding humor, proverbs, or metaphors — NO / YES

If YES, how much did you experience this as a problem over the last month?

Not at all 0 1 2 3 4 5 6 7 8 9 10 *Extremely*

m. Ever have difficulty **communicating a message concisely and clearly**, such as telling the point of a story — NO / YES

If YES, how much did you experience this as a problem over the last month?

Not at all 0 1 2 3 4 5 6 7 8 9 10 *Extremely*

n. Ever have difficulty **explaining emotions or feelings** — NO / YES

If YES, how much did you experience this as a problem over the last month?

Not at all 0 1 2 3 4 5 6 7 8 9 10 *Extremely*

o. Ever suffer from **unclear speech**, such as lisping or stuttering — NO / YES

If YES, how much did you experience this as a problem over the last month?

Not at all 0 1 2 3 4 5 6 7 8 9 10 *Extremely*

p. Ever suffer from **repeating words or phrases** over and over again — NO / YES

If YES, how much did you experience this as a problem over the last month?

Not at all 0 1 2 3 4 5 6 7 8 9 10 *Extremely*

q. Ever have difficulty with **poor eye contact** — NO / YES

If YES, how much did you experience this as a problem over the last month?

Not at all 0 1 2 3 4 5 6 7 8 9 10 *Extremely*

r. Ever suffer from **repetitive behavior**, such as doing the same thing over and over again — NO / YES

If YES, how much did you experience this as a problem over the last month?

Not at all 0 1 2 3 4 5 6 7 8 9 10 *Extremely*

s. Ever suffer from **rigid or inflexible behavior**, such as always preferring to do things the same way, difficulty with unexpected changes, or difficulty changing opinions — NO / YES

If YES, how much did you experience this as a problem over the last month?

Not at all 0 1 2 3 4 5 6 7 8 9 10 *Extremely*

t. Ever shown **age-inappropriate behavior**, such as not responding as expected for your/their age or developmental stage — NO / YES

If YES, how much did you experience this as a problem over the last month?

Not at all 0 1 2 3 4 5 6 7 8 9 10 *Extremely*

u. Ever have difficulty **interacting with peers**, such as difficulty with making friends, avoiding contact, bullying or being bullied, being excluded, or having frequent arguments — NO / YES

If YES, how much did you experience this as a problem over the last month?

Not at all 0 1 2 3 4 5 6 7 8 9 10 *Extremely*

Did [you/the person with KS]:

v. Ever suffer from **hyperactivity**, such as having lots of energy, or moving or talking all the time — NO / YES

If YES, how much did you experience this as a problem over the last month?

Not at all 0 1 2 3 4 5 6 7 8 9 10 *Extremely*

w. Ever have difficulty **paying attention or concentrating** — NO / YES

If YES, how much did you experience this as a problem over the last month?

Not at all 0 1 2 3 4 5 6 7 8 9 10 *Extremely*

x. Ever suffer from **restless behavior**, such as not being able to sit still, fidgeting with hands, or finding it difficult to relax — NO / YES

If YES, how much did you experience this as a problem over the last month?

Not at all 0 1 2 3 4 5 6 7 8 9 10 *Extremely*

y. Ever suffer from **impulsive behavior**, such as interrupting a conversation, having difficulty waiting turns, or making decisions without thinking about the possible consequences — NO / YES

If YES, how much did you experience this as a problem over the last month?

Not at all 0 1 2 3 4 5 6 7 8 9 10 *Extremely*

z. Ever have difficulty with **eating**, such as eating much or little, eating unusual things or eating day and night — NO / YES

If YES, how much did you experience this as a problem over the last month?

Not at all 0 1 2 3 4 5 6 7 8 9 10 *Extremely*

aa. Ever suffer from **sleep problems**, such as often going to sleep late, having trouble falling asleep or waking up at night — NO / YES

If YES, how much did you experience this as a problem over the last month?

Not at all 0 1 2 3 4 5 6 7 8 9 10 *Extremely*

bb. Ever have problems with **addiction**, such as difficulty stopping gaming or drinking too much alcohol, smoking or using drugs — NO / YES

If YES, how much did you experience this as a problem over the last month?

Not at all 0 1 2 3 4 5 6 7 8 9 10 *Extremely*

cc. Ever experience **other difficulties** in terms of behavior? — NO / YES

If YES, note below and explain how much of a problem this was over the last month.

If you answered YES to any of the items in question 2:

Did [you/the person with KS] receive further evaluation or counseling for these behavioral difficulties? — NO / YES

Would you like further evaluation or guidance for [yourself/the person with KS]? — NO / YES

Notes:

Question 3: Intellectual ability

a. Have you ever worried about your **intellectual ability**/the intellectual ability of the person with KS? NO YES

b. Did [you/the person with KS] ever have a **formal intelligence assessment** using an IQ test? NO YES

If YES, what were the results (cross)

- Above average intelligence (IQ>115)
- Average intelligence (IQ 85 - 115)
- Bordeline intelligence (IQ 70 – 84)
- Mild intellectual disablity (IQ 50-69)
- Moderate intellectual disablity (IQ 36-49)
- Severe intellectual disablity (IQ 20-35)
- Profound intellectual disablity (IQ <20)
- I don't know

If NO, how would you rate the intellectual ability of [yourself/the person with KS] (cross)

- Above average intelligence
- Average intelligence
- Borderline intelligence
- Intellectual disablity

Would you like further evaluation or guidance for [yourself/the person with KS]? NO YES

Notes (any comments or results from the IQ test)

Question 4: Neuropsychological abilities

Did [you/the person with KS]:

a. Ever have difficulty with **memory**, such as difficulty remembering instructions just given, recent events or experiences from the past NO YES

If YES, how much did you experience this as a problem over the last month?

Not at all 0 1 2 3 4 5 6 7 8 9 10 *Extremely*

b. Ever have difficulty with **attention**, such as being easily distracted, forgetting things quickly, difficulty completing tasks, or daydreaming NO YES

If YES, how much did you experience this as a problem over the last month?

Not at all 0 1 2 3 4 5 6 7 8 9 10 *Extremely*

c. Ever have difficulty **performing multiple tasks at once**, such as doing multiple things at once, e.g., cooking and watching television, or holding a conversation while driving a car or riding a bicycle NO YES

If YES, how much did you experience this as a problem over the last month?

Not at all 0 1 2 3 4 5 6 7 8 9 10 *Extremely*

d. Ever have difficulty with **visual-spatial skills**, such as doing puzzles, drawing something after, or assembling furniture NO YES

If YES, how much did you experience this as a problem over the last month?

Not at all 0 1 2 3 4 5 6 7 8 9 10 *Extremely*

e. Ever have difficulty with **executive skills**, such as planning or organizing tasks and activities, starting or completing tasks independently, or estimating tasks and adjusting if something goes wrong — NO / YES

If YES, how much did you experience this as a problem over the last month?

Not at all 0 1 2 3 4 5 6 7 8 9 10 *Extremely*

f. Ever have difficulty with **orientation in time and space**, such as estimating the time needed for a particular task, knowing what day it is, knowing one's location, or traveling by train or bus — NO / YES

If YES, how much did you experience this as a problem over the last month?

Not at all 0 1 2 3 4 5 6 7 8 9 10 *Extremely*

g. Ever have difficulty with **motor skills,** such as having difficulty writing legibly, catching or throwing a ball, being clumsy, often tripping or falling — NO / YES

If YES, how much did you experience this as a problem over the last month?

Not at all 0 1 2 3 4 5 6 7 8 9 10 *Extremely*

h. Ever have difficulty with **processing speed**, such as being slower to complete tasks or having a slower response because more time is needed to process information — NO / YES

If YES, how much did you experience this as a problem over the last month?

Not at all 0 1 2 3 4 5 6 7 8 9 10 *Extremely*

i. Ever have difficulty with **social cognition**, such as difficulty recognizing and understanding, and/or empathizing with emotions of others — NO / YES

If YES, how much did you experience this as a problem over the last month?

Not at all 0 1 2 3 4 5 6 7 8 9 10 *Extremely*

j. Are there any other difficulties you would like to mention here? — NO / YES

If YES, note below and explain how much of a problem this was over the last month.

If you answered YES to any of the items in question 4:

Did [you/the person with KS] receive further evaluation or counseling for this? — NO / YES

Would you like further evaluation or guidance for [yourself/the person with KS]? — NO / YES

Notes:

Question 5: Psychiatric Disorders

Did [you/the person with KS] ever receive an official diagnosis from a health care provider, such as:

a. Autism Spectrum Disorder (ASD) — NO / YES

b. Attention Deficit Hyperactivity Disorder (ADHD) — NO / YES

c. Depressive disorder — NO / YES

d. Anxiety disorder, including panic, phobia, separation anxiety disorder — NO / YES

e. Obsessive Compulsive Disorder (OCD) — NO / YES

f. Psychotic disorder, including schizophrenia, delusions, or hallucinations — NO / YES

g. Other psychiatric disorder(s). If YES, note below. — NO / YES

If you answered YES to any of the items in question 5:

Did [you/the person with KS] receive further evaluation or counseling for this? ☐ NO ☐ YES

Would you like further evaluation or guidance for [yourself/ the person with KS]? ☐ NO ☐ YES

Notes:

Question 6: Academic skills

What is [your/the person with KS'] **level of education**?

- ☐ Not yet going to school
- ☐ Preschool education
- ☐ Primary education
- ☐ Secondary education
- ☐ Special education
- ☐ Higher education (university or college)
- ☐ Other: ______

Did [you/the person with KS]:

a. Ever have difficulty with **reading**? ☐ NO ☐ YES ☐ NOT YET IN SCHOOL

If YES, how much did you experience this as a problem over the last month?

Not at all 0 ☐ 1 ☐ 2 ☐ 3 ☐ 4 ☐ 5 ☐ 6 ☐ 7 ☐ 8 ☐ 9 ☐ 10 ☐ *Extremely*

b. Ever have difficulty with **writing**? ☐ NO ☐ YES ☐ NOT YET IN SCHOOL

If YES, how much did you experience this as a problem over the last month?

Not at all 0 ☐ 1 ☐ 2 ☐ 3 ☐ 4 ☐ 5 ☐ 6 ☐ 7 ☐ 8 ☐ 9 ☐ 10 ☐ *Extremely*

c. Ever have difficulty with **spelling**? ☐ NO ☐ YES ☐ NOT YET IN SCHOOL

If YES, how much did you experience this as a problem over the last month?

Not at all 0 ☐ 1 ☐ 2 ☐ 3 ☐ 4 ☐ 5 ☐ 6 ☐ 7 ☐ 8 ☐ 9 ☐ 10 ☐ *Extremely*

d. Ever have difficulty with **mathematics**? ☐ NO ☐ YES ☐ NOT YET IN SCHOOL

If YES, how much did you experience this as a problem over the last month?

Not at all 0 ☐ 1 ☐ 2 ☐ 3 ☐ 4 ☐ 5 ☐ 6 ☐ 7 ☐ 8 ☐ 9 ☐ 10 ☐ *Extremely*

e. Ever have **other learning difficulties** at school? ☐ NO ☐ YES ☐ NOT YET IN SCHOOL

If YES, note below and explain how much of a problem this was over the last month.

Did [you/the person with KS] ever receive a diagnosis of a **learning disability** such as dyslexia (difficulty with reading and writing) or dyscalculia (difficulty with math and numbers)? ☐ NO ☐ YES

If you answered YES to any of the items in question 6:

Did [you/the person with KS] ever receive additional evaluation, additional guidance, or a modified track at school to address these difficulties? ☐ NO ☐ YES

Would you like further evaluation or guidance (e.g., help from a care teacher, school doctor, speech therapist, etc.) for [you/the person with KS]? ☐ NO ☐ YES

Notes:

Question 7: Self-sufficiency

To what extent can [you/the person with KS] take care of [yourself/themselves] in the following domains?

In the domain of

a. **Self-care**: personal hygiene (dressing, washing, grooming), monitoring own health (going to the doctor, taking medication) — No problem / Help needed / Not applicable

b. **Finances**: earning money, saving, paying bills — No problem / Help needed / Not applicable

c. **Daily living:** living independently, grocery shopping, household chores (cleaning, washing clothes, doing the dishes) — No problem / Help needed / Not applicable

d. **Social network**: getting enough support from family, friends, doctor/psychologist and others — No problem / Help needed / Not applicable

Notes:

Question 8: Psychosocial functioning

Aside from the issues described above, KS can impact the psychosocial functioning or well-being of people with KS and those around them.

8.1 Social skills and relationships

Did [you/the person with KS]:

a. Ever have difficulty with **social interactions**, such as struggling to interact with peers or adults — NO / YES

If YES, how much did you experience this as a problem over the last month?

Not at all 0 1 2 3 4 5 6 7 8 9 10 *Extremely*

b. Ever experience **social problems**, such as bullying, discrimination, exclusion from social groups or activities, inadequate access to care or education, or social isolation — NO / YES

If YES, how much did you experience this as a problem over the last month?

Not at all 0 1 2 3 4 5 6 7 8 9 10 *Extremely*

c. Ever have difficulty forming, maintaining, or deepening relationships, such as:

ii. **Friendships** — NO / YES

If YES, how much did you experience this as a problem over the last month?

Not at all 0 1 2 3 4 5 6 7 8 9 10 *Extremely*

ii. **Romantic relationships** — NO / YES

If YES, how much did you experience this as a problem over the last month?

Not at all 0 1 2 3 4 5 6 7 8 9 10 *Extremely*

8.2 Body perception

a. Ever worry about **body image, i.e.**, how you look at your own body — NO / YES

If YES, how much did you experience this as a problem over the last month?

Not at all 0 1 2 3 4 5 6 7 8 9 10 *Extremely*

Did [you/the person with KS]:

b. Ever worry about **physical problems**, e.g., weight gain, breast formation, reduced bone strength — NO — YES

If YES, how much did you experience this as a problem over the last month?

Not at all 0 1 2 3 4 5 6 7 8 9 10 *Extremely*

c. Ever suffer from **low energy or fatigue** — NO — YES

If YES, how much did you experience this as a problem over the last month?

Not at all 0 1 2 3 4 5 6 7 8 9 10 *Extremely*

d. Ever worry about **fertility** — NO — YES

If YES, how much did you experience this as a problem over the last month?

Not at all 0 1 2 3 4 5 6 7 8 9 10 *Extremely*

e. Ever worry about **sexual problems**, such as low or high sexual interest — NO — YES

If YES, how much did you experience this as a problem over the last month?

Not at all 0 1 2 3 4 5 6 7 8 9 10 *Extremely*

f. Ever have concerns about or doubt your/their **gender identity** such as being born male, but not/only partially identifying with it — NO — YES

If YES, how much did you experience this as a problem over the last month?

Not at all 0 1 2 3 4 5 6 7 8 9 10 *Extremely*

8.3 Emotional well-being

a. Ever suffer from **low self-esteem** — NO — YES

If YES, how much did you experience this as a problem over the last month?

Not at all 0 1 2 3 4 5 6 7 8 9 10 *Extremely*

b. Ever worry about your/their **mental health** — NO — YES

If YES, how much did you experience this as a problem over the last month?

Not at all 0 1 2 3 4 5 6 7 8 9 10 *Extremely*

c. Ever experience **negative emotions** such as anger, sadness, or stress because of the diagnosis of KS — NO — YES

If YES, how much did you experience this as a problem over the last month?

Not at all 0 1 2 3 4 5 6 7 8 9 10 *Extremely*

d. Ever suffer from **compulsive thoughts** (recurrent, unwanted, and uncontrollable thoughts) — NO — YES

If YES, how much did you experience this as a problem over the last month?

Not at all 0 1 2 3 4 5 6 7 8 9 10 *Extremely*

e. Ever have thoughts of **hurting onself or ending your/their life** — NO — YES

****If YES, we encourage you to contact your physician or a health care provider***

If YES, how much was this as a problem over the last month?

Not at all 0 1 2 3 4 5 6 7 8 9 10 *Extremely*

8.4 Impact of KS in different life contexts such as family and professional contexts

Did you (the person completing the checklist):

a. Ever experience an impact of KS on your **family life**, such as a lot of stress, tension, arguments, or adjustments in the family — NO / YES

If YES, how much did you experience this as a problem over the last month?

Not at all 0 1 2 3 4 5 6 7 8 9 10 *Extremely*

b. Ever experience difficulties in the **relationship between parent and child** because of the impact of KS — NO / YES

If YES, how much did you experience this as a problem over the last month?

Not at all 0 1 2 3 4 5 6 7 8 9 10 *Extremely*

c. Ever experience difficulties in the **relationship between parents** because of the impact of KS — NO / YES

If YES, how much did you experience this as a problem over the last month?

Not at all 0 1 2 3 4 5 6 7 8 9 10 *Extremely*

d. Ever felt that your **family** was **isolated** from others outside the family because of the impact of KS — NO / YES

If YES, how much did you experience this as a problem over the last month?

Not at all 0 1 2 3 4 5 6 7 8 9 10 *Extremely*

e. Ever experience an impact of KS on your **professional life** such as difficulty finding a job, performing a job, interacting with co-workers or other job-related issues — NO / YES

If YES, how much did you experience this as a problem over the last month?

Not at all 0 1 2 3 4 5 6 7 8 9 10 *Extremely*

If you answered YES to any of the items in question 8:

Did you and/or your family receive further evaluation or support for this? — NO / YES

Would you like further evaluation or guidance for any of these psychosocial problems, such as psychosocial support, career counseling, consultation with a physician or psychologist, etc.? — NO / YES

Notes:

Question 9: Assessment of the impact of KS

Looking at all the issues from the checklist we just went through. To what extent have these difficulties affected, worried or stressed [you/the person with KS]?

Not at all 0 1 2 3 4 5 6 7 8 9 10 *Extremely*

Question 10: Additional concerns

Are there any other concerns regarding KAND that were not adressed in this checklist? ☐ NO ☐ YES

If YES, note below

Question 11: Priority list

Of all the concerns listed above, which are the most important for you to work on?

What topics related to KS would you like more information on?

Question 12: Strategies

We all develop **ways to cope with KAND in everyday life**. Please write down what strategies you use (as a parent of someone with KS or a person with KS yourself) to deal with the challenges you face. This can be helpful when monitoring progress over time.

Question 13: Strengths and positive traits

So far we have focused on difficulties and challenges. However, each person with KS also has their own strengths, skills and talents! Write down some **strengths or positive traits** here, thinking particularly about the last month. This may include happy moments, small victories, or anything else that you celebrate (or that makes you happy).

